# Vertical distribution of soil available phosphorus and soil available potassium in the critical zone on the Loess Plateau, China

**DOI:** 10.1038/s41598-021-82677-4

**Published:** 2021-02-04

**Authors:** Hanyang Tian, Jiangbo Qiao, Yuanjun Zhu, Xiaoxu Jia, Ming’an Shao

**Affiliations:** 1 State Key Laboratory of Soil Erosion and Dryland Farming on the Loess Plateau, Institute of Soil and Water Conservation, Chinese Academy of Sciences and Ministry of Water Resources, Yangling, 712100 China; 2 State Key Laboratory of Soil Erosion and Dryland Agriculture on the Loess Plateau, Northwest A&F University, Yangling, 712100 China; 3 Key Laboratory of Ecosystem Network Observation and Modeling, Institute of Geographic Sciences and Natural Resources Research, Chinese Academy of Sciences, Beijing, 100101 China; 4 University of Chinese Academy of Sciences, Beijing, 100101 China

**Keywords:** Ecology, Environmental sciences

## Abstract

Soil available phosphorus (SAP) and soil available potassium (SAK) are important elements in the growth of plants. However, limited data are available regarding the vertical distribution of SAP and SAK in deep soil profiles. In this study, we investigated the vertical variations in SAP and SAK in the critical zone on the Loess Plateau (50–200 m), China, by using classical statistical and geostatistical methods. The soil samples were collected from the top of the soil profile down to the bedrock by soil core drilling at five typical sites. SAP decreased throughout the profile. Whereas the SAK exhibited an increasing trend at all sites. The mean SAP concentration ranged from 0.94 to 32.56 mg kg^–1^ at the sampling sites and the SAK concentration ranged from 44.51 to 229.31 mg kg^–1^. At all of the sampling sites, SAK was significantly positively correlated with the depth and clay content, but there was a significantly negative correlation between the SAK and the sand content. The exponential model could fit most variograms of SAP and SAK at all sampling sites. The results obtained in this study to improve our comprehension of the SAP or SAK distribution conditions on the Loess Plateau, which is important for reasonable fertilizer application and vegetation planting practices.

## Introduction

Phosphorus (P) and potassium (K) are the main nutrient elements in plants. Among them, soil available P (SAP) and soil available K (SAK) can be directly used by plants. Therefore, SAP and SAK are effective indicators to judge soil fertility and plant growth. Determining the spatial distribution of SAP and SAK as well as the factors that influence them are important for implementing reasonable vegetation planting and fertilizer application practices.

The spatial distribution of SAP and SAK and their related factors had been studied widely at different scales. For example, Li et al.^1^ evaluated the spatial variations in the SAP and SAK in the Huanghuadianzi watershed. In addition, Bogunovic et al.^2^ investigated the spatial distribution of soil chemical properties on an organic farm in Croatia. Chen et al.^3^ determined the characteristic distribution of the SAP and SAK in farmland in Lanzhou, China. However, most previous studies focused on the spatial distribution of the SAP and SAK in the upper soil layer (< 1 m), few have considered their spatial distribution in deep soil layers.

The Loess Plateau has deep loess deposits that range in thickness from 30 to 200 m^4^ in China. Due to the low content of the soil nutrient and the groundwater depth of 50–100 m^5^, the formation and loss of deep soil nutrients caused by the participation of deep soil moisture in the water cycle have a certain impact on the biogeochemical cycle. For a long time, few studies had shown that the nutrient content of deep soil changes, most of which were concentrated in the surface layer of soil, it is very meaningful to study the changes in SAP and SAK contents in deep profiles.

The objectives of this study were: (1) to investigate the vertical distributions of the SAP and SAK in the critical zone on the Loess Plateau; (2) to evaluate the factors that influence the SAP and SAK distributions; and (3) to evaluate the spatial variations in SAP and SAK.

## Materials and methods

### Study area

**Figure 1:**
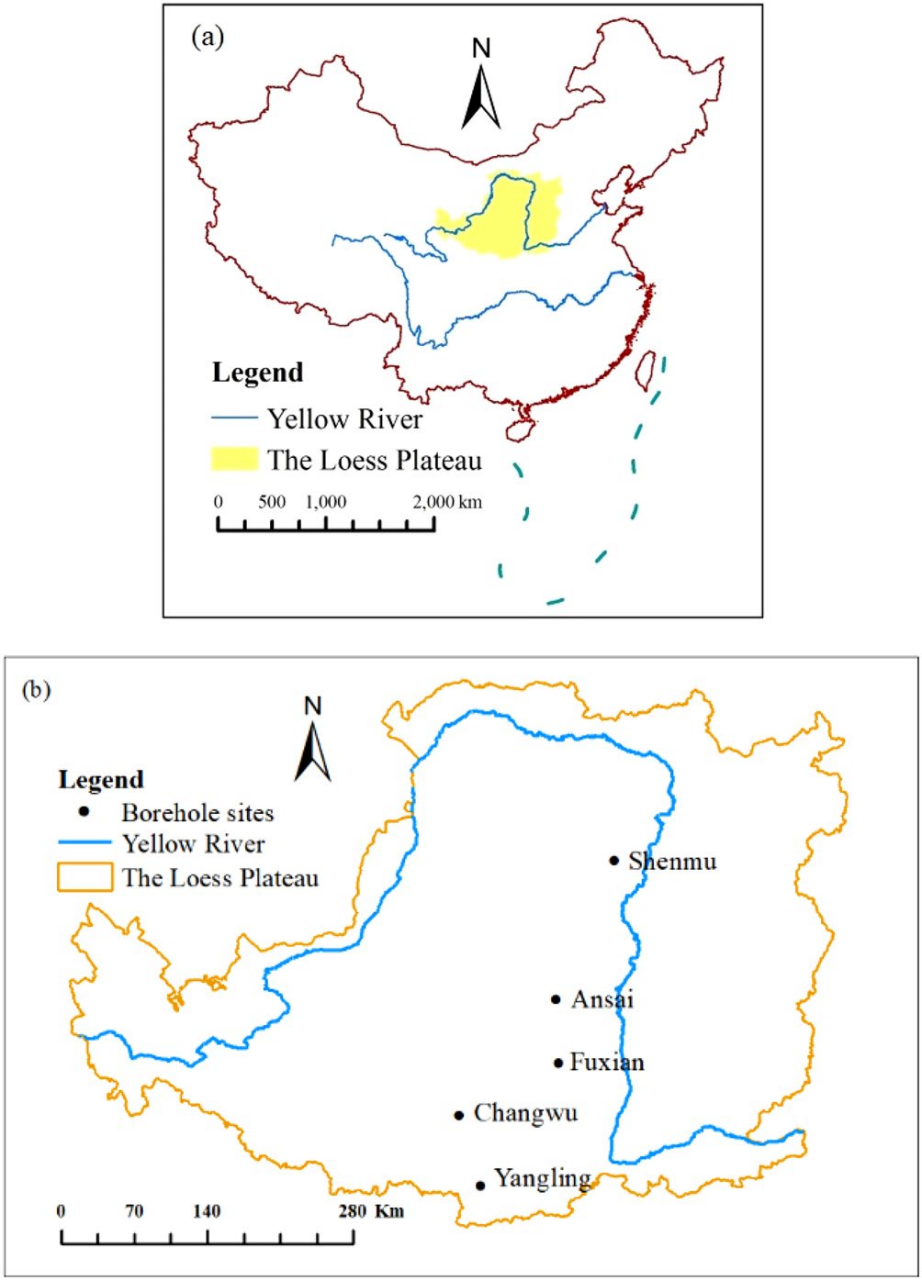
Locations of the Loess Plateau region in China (**a**) and the sampling sites (**b**); image data processed by ArcGIS 10.5 http://developers.arcgis.com.

The study was conducted across the Loess Plateau (33°43′–41°16′N, 100°54′–114°33′E) (Fig. 1a), which represents approximately 6.5% of the total area of China^6^. The study area is dominated by temperate, arid, and semiarid continental monsoon climates. The annual evaporation is 1400–2000 mm, and the annual temperature ranges from 3.6 °C in the northwest to 14.3 °C in the southeast on the Loess Plateau^7^, while the annual precipitation ranges from 150 to 800 mm, where 55–78% of the precipitation falls from June to September^7^. The annual solar radiation ranges from 5.0 × 10^9^ to 6.7 × 10^9^ J m^−2^. The vegetation zones are forest, forest-steppe, typical-steppe, desert-steppe, and steppe-desert zones^8^ from southeast to northwest.

### Field sampling

According to the different climate zones and vegetation types, five classic sampling sites were selected (Fig. 1b) on the Loess Plateau, which were Yangling, Changwu, Fuxian, Ansai, and Shenmu from south to north. Drilling equipment (assembled by Xi’an Qinyan Drilling Co. Ltd, China) was used to collect soil samples from soil surface down to bedrock. At each sampling site, disturbed soil samples were collected to determine the SAP and SAK concentrations, pH, soil particle composition, and soil organic matter contents. In addition, disturbed soil samples were collected from the middle of the soil column at 1-m intervals (i.e., 0.5 m, 1.5 m, 2.5 m, 3.5 m, etc.). The drilling and sampling work was carried out from April 28 to June 28, 2016. The total numbers of disturbed soil samples collected from Yangling, Changwu, Fuxian, Ansai, and Shenmu were 103, 205, 181, 161, and 58, respectively, and the corresponding soil drilling depths were 103.5 m, 204.5 m, 187.5 m, 161.6 m, and 56.6 m, respectively.

### Laboratory analyses

Undisturbed soil samples were air-dried, separated, and passed through 0.25-mm or 2-mm sieves. SAP and SAK were extracted with ammonium lactate solution and detected by spectrophotometry and flame photometry. Soil total nitrogen (STN) concentrations were determined by the Kjeldahl digestion procedure^9^. Soil total phosphorus (STP) concentrations were determined by molybdenum antimony blue colorimetry^10^. The soil organic carbon (SOC) contents were analyzed by dichromate oxidation method^11^. The soil particle composition was determined by laser diffraction (Mastersizer 2000, Malvern Instruments, Malvern, UK)^12^. According to the mixture of soil and water mass ratio of 1:1, the pH value was determined with a pH meter equipped with a calibrated combined glass electrode. The soil water content (SWC) was determined by the mass loss after drying to constant mass in an oven at 105 °C^13^. The calcium carbonate content was determined by the acid-neutralization method^14^.

### Geostatistical analysis

The geostatistical analysis was chosen to determine the spatial structure of the spatially dependent soil properties^15^, where a semivariogram was employed to quantify the spatial patterns of the variables. The equation for the semivariogram is^16^:

1 $$ {\text{R}}\left( {\text{h}} \right) \, = \frac{1}{{2{\text{N}}\left( {\text{h}} \right)}}\mathop \sum \limits_{{{\text{i}} = 1}}^{{{\text{N}}\left( {\text{h}} \right)}} \left[ {{\text{Z}}\left( {{\text{x}}_{{\text{i}}} } \right){-}{\text{Z}}\left( {{\text{x}}_{{{\text{i}} + {\text{h}}}} } \right)} \right]^{{2}} , $$

where for each site i, N(h) is the number of pairs separated by h, and Z(x_i_) is the value at location xi and Z(x_i+h_) for x_i+h_. There are four semivariogram models (spherical, exponential, linear, and Gaussian), which can be employed to describe the semivariogram, and the best fitting model is selected according to the smallest residual sum of squares (RSS) and the largest coefficient of determination (R^2^). The equation of each semivariogram model is^16^:

Exponential model:

2 $$ {\text{R}}\left( {\text{h}} \right) = {\text{C}}_{0} + {\text{C}}\left[ {({1}{-}{\text{exp}}( - {\text{h}}/{\text{A}}_{0} )} \right] $$

Linear Model:

3 $$ {\text{R}}\left( {\text{h}} \right) = {\text{C}}_{0} + \left[ {{\text{h}}\left( {{\text{C}}/{\text{A}}_{0} } \right)} \right] $$

Spherical Model:

4 $$ {\text{R}}\left( {\text{h}} \right) = {\text{C}}0 + {\text{C}}\left[ {{1}.{5}\left( {{\text{h}}/{\text{A}}_{0} } \right) - 0.{5}\left( {{\text{h}}/{\text{A}}_{0} } \right)^{{3}} } \right] \;\;\;\;\;\;\;\;\; {\text{h}} \le {\text{A}}0 $$

5 $$ {\text{R}}\left( {\text{h}} \right) = {\text{C}}_{0} + {\text{C}}\;\;\;\;\;\;\;\;\;\;{\text{h}} \ge {\text{A}}0 $$

Gaussian Model:

6 $$ {\text{R}}\left( {\text{h}} \right) = {\text{C}}_{0} + {\text{C}}\left[ {{1} - {\text{exp}}\left( { - {\text{h}}^{{2}} /{\text{A}}_{0}^{{2}} } \right)} \right] $$

where C_0_ indicates the nugget value, which is the short-range structure that occurs at distances smaller than the sampling interval, microheterogeneity, and experimental error; C_0_ + C is the sill indicating the random and structural variation, and; A_0_ is the range indicating the spatial correlation at different distances.

### Statistical analysis

Descriptive statistical analyses (maximum, minimum, average, and coefficient of variation), Pearson’s correlation analysis, and linear regression analysis was performed with SPSS 16.0 (IBM SPSS, Chicago, IL, USA). Geostatistical analysis was performed with GS + software (version 7.05).

## Results

### Vertical distributions of SAP and SAK

**Figure 2:**
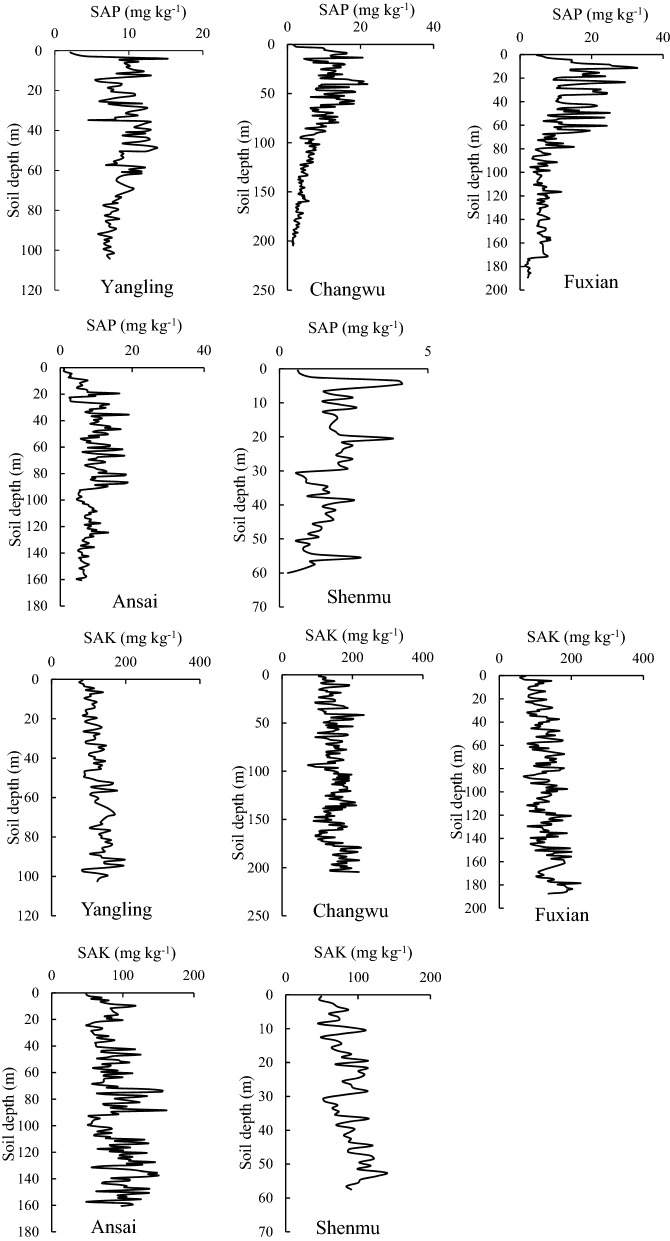
Vertical distributions of SAP and SAK concentrations at different sampling sites; the data extracted from GS + and mapped by Excel https://www.microsoft.com/zh-cn/microsoft-365/excel.

Figure 2 shows the vertical distribution of the SAP and SAK contents at all of the sampling sites. Trends in the SAP decreased among the different sampling sites, whereas the SAK concentration increased gradually with depth at all of the sampling sites.

Table 1 shows the descriptive statistics obtained for SAP and SAK at the different sampling sites. SAP ranged from 0.28 to 32.56 mg kg^–1^ at the sampling sites and SAK ranged from 44.51 to 229.31 mg kg^–1^. The CV values of SAP and SAK ranged from 0.26 to 0.67 and from 0.21 to 0.30, respectively, and the SAP and SAK concentrations exhibited moderate variation at different sampling sites^17^. In addition, the mean SAP and SAK concentrations tended to decrease from south to north among the sampling sites.

**Table 1:** Descriptive statistics for the soil available phosphorus (SAP) and soil available potassium (SAK) concentrations at different sampling sites.

SS	SCP	No	Min	Max	Mean	CV
Yangling	SAP	103	2.10	15.19	9.03	0.26
	SAK	103	58.78	197.80	122.34	0.21
Changwu	SAP	205	2.28	21.69	10.60	0.38
	SAK	205	74.07	229.31	149.01	0.21
Fuxian	SAP	181	1.39	32.56	9.42	0.67
	SAK	181	57.81	225.83	127.62	0.26
Ansai	SAP	161	0.94	19.10	8.39	0.43
	SAK	161	48.51	161.29	88.15	0.30
Shenmu	SAP	58	0.28	4.45	3.64	0.25
	SAK	58	44.51	139.17	86.16	0.25

*SS* sampling site, *SCP* soil chemical property, *No.* Number, *Min* minimum, *Max* maximum, *CV* coefficient of variation, *SAP* soil available phosphorus, *SAK* soil available potassium.

### Correlation analysis

Pearson's correlation coefficients were calculated to determine the strengths of the possible relationships between SAP and SAK with the other basic soil properties. Table 2 shows the Pearson's correlation coefficients obtained between SAP and SAK, and the selected soil properties.

**Table 2:** Pearson's correlation coefficients between the soil available phosphorus (SAP), soil available potassium (SAK), and selected soil properties.

SCP	Yangling		Changwu		Fuxian		Ansai		Shenmu	
	SAP	SAK	SAP	SAK	SAP	SAK	SAP	SAK	SAP	SAK
Depth	– 0.25*	0.54**	– 0.55**	0.24**	– 0.70**	0.46**	– 0.05	0.39**	0.12	0.56**
SAK	– 0.16	1	– 0.27**	1	– 0.39**	1	0.09	1	0.24	1
SAP	1	– 0.16	1	– 0.27**	1	– 0.39**	1	0.09	1	0.24
SWC	0.16	– 0.13	0.48**	– 0.32**	– 0.27**	0.18*	0.41**	0.26**	0.22	0.54**
Sand	0.004	– 0.30**	– 0.02	– 0.49**	0.15*	– 0.42**	– 0.14	– 0.64**	– 0.25	– 0.48**
Silt	– 0.06	– 0.26*	0.38**	– 0.31**	0.05	– 0.15*	0.12	0.45**	0.21	0.43**
Clay	0.04	0.33**	– 0.27**	0.58**	– 0.17*	0.49**	0.1	0.61**	0.21	0.36**
STP	0.29**	– 0.25*	0.14*	– 0.35**	0.29**	– 0.51**	– 0	– 0.23**	– 0.03	– 0.18
STN	– 0.18	– 0.08	0.1	0.16*	0.16*	0.01	0.12	0.45**	0.26*	0.50**
SOC	– 0.03	– 0.36**	0.11	– 0.006	0.21**	– 0.19**	– 0.01	– 0.007	0.04	– 0.18
pH	– 0.12	0.16	0.22**	0.03	– 0.35**	0.29**	– 0.14	0.15	– 0.22	– 0.58**
CaCO_3_	0.30**	– 0.49**	– 0.23**	– 0.24**	– 0.06	– 0.14	0.05	– 0.61**	0.15	– 0.12

*SCP* soil chemical properties, *SWC* soil water content, *STN* soil total nitrogen, *STP* soil total phosphorus, *SOC* soil organic carbon, *SAP* soil available phosphorus, *SAK* soil available potassium.

*Correlation is significant at the 0.05 level.

**Correlation is significant at the 0.01 level.

The correlation analysis results obtained for SAP varied among the different sampling sites. For example, SAP had significant correlations with most of the selected variables (except for sand, STN, and SOC) at Changwu, but it was only significantly correlated with SWC at Ansai. Similar to SAP, the correlation analysis results varied for SAK at different sampling sites. At all of the sampling sites, SAK had significant positive correlations with the depth and clay content and significant negative correlations with the sand content.

### Stepwise multiple linear regression analysis (SMLRA)

Table 3 shows the results obtained by SMLRA between SAP, SAK, and the significantly correlated factors, which were conducted to determine the contributions of these independent variables to the variations in SAP or SAK. The adjusted r^2^ values for SAP ranged from 5.2 to 54.9%, and those for SAK ranged from 44.6% to 64.3%, thereby indicating that the selected soil properties explained more of the variation in SAK than SAP. For SAP, the adjusted r^2^ values obtained at Yangling, Changwu, Fuxian, Ansai, and Shenmu were 7.8%, 32.6%, 54.9%, 16.3%, and 5.2%, respectively, and 49.5%, 49%, 44.6%, 59%, and 64.3% for SAK. In addition, the independent variables that explained most of the variations in SAP at Yangling, Changwu, Fuxian, Ansai, and Shenmu were CaCO_3_ (7.8%), depth (30.4%), depth (48.9%), SWC (16.3%), and STN (5.2%), respectively, and depth (28.4), clay (33.1%), STP (25.4%), clay (37.3%), and depth (29.8%) for SAK.

**Table 3:** Results obtained by multiple stepwise regression analyses between SAP, SAK, and the significantly correlated factors.

SS	DV	IV	Coefficient	SE	P	EV	Adjusted r^2^
Yangling	SAP	Constant	8.098	0.408	0.000		0.078
		CaCO_3_	0.015	0.005	0.003	0.078	
	SAK	Constant	66.549	14.753	0.000		
		Depth	0.399	0.063	0.000	0.284	0.495
		CaCO_3_	– 0.172	0.040	0.000	0.143	
		Clay	1.655	0.452	0.000	0.067	
Changwu	SAP	Constant	19.470	1.072	0.000		0.326
		Depth	– 0.051	0.005	0.000	0.304	
		SWC	– 2.402	0.467	0.000	0.022	
	SAK	Constant	178.258	24.913	0.000		0.490
		Clay	2.187	0.438	0.000	0.331	
		CaCO_3_	– 0.267	0.039	0.000	0.101	
		SAP	– 1.372	0.455	0.003	0.033	
		Sand	– 1.424	0.557	0.011	0.011	
		SWC	– 1.708	0.670	0.012	0.014	
Fuxian	SAP	Constant	– 53.126	23.550	0.025		0.549
		Depth	– 0.114	0.009	0.000	0.489	
		STN	– 23.894	6.265	0.000	0.035	
		pH	9.063	2.781	0.001	0.025	
	SAK	Constant	125.460	22.385	0.000		0.446
		STP	– 107.268	19.443	0.000	0.254	
		Clay	0.947	0.544	0.083	0.097	
		SWC	1.753	0.590	0.003	0.058	
		Depth	0.133	0.042	0.002	0.024	
		Sand	– 1.259	0.565	0.027	0.013	
Ansai	SAP	Constant	2.976	0.988	0.003		0.163
		SWC	0.314	0.055	0.000	0.163	
	SAK	Constant	50.927	8.696	0.000		0.590
		Clay	1.616	0.364	0.000	0.373	
		CaCO_3_	– 0.259	0.039	0.000	0.179	
		STN	123.572	31.939	0.000	0.018	
		Depth	0.108	0.037	0.004	0.020	
Shenmu	SAP	Constant	4.107	0.808	0.000		0.052
		STN	13.051	6.433	0.047	0.052	
	SAK	Constant	3.531	8.990	0.696		0.643
		Depth	0.836	0.104	0.000	0.298	
		STN	476.166	64.153	0.000	0.345	

*SS* sampling sites, *DV* dependent variable, *IV* independent variable, *SE* standard error, *EV* explained variation, *P* significance level.

### Spatial structure of the SAP and SAK

**Figure 3:**
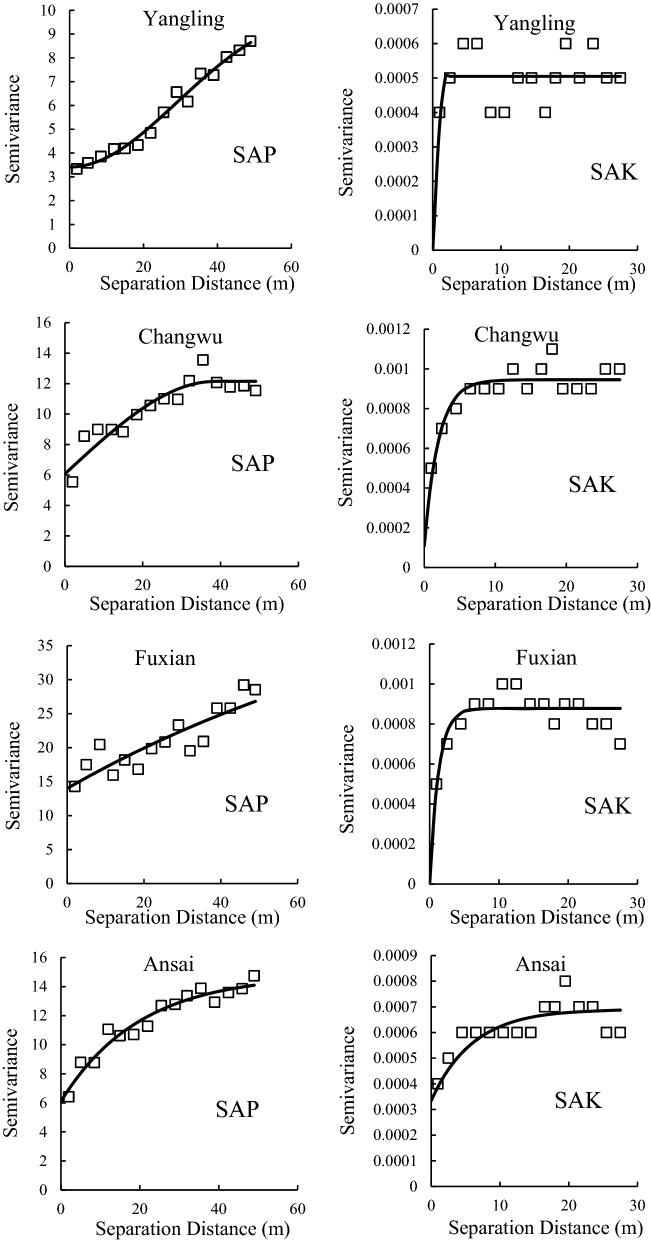

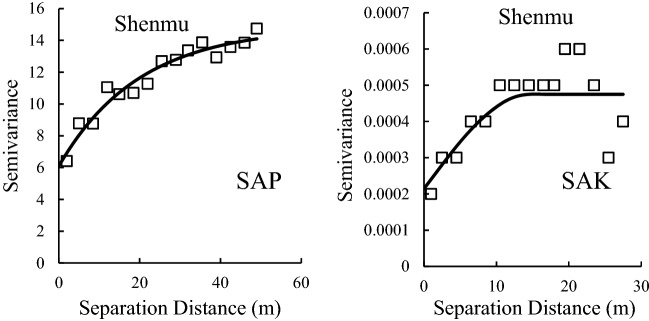
Semivariograms of soil available phosphorus (SAP) and soil available potassium for the sampling sites; the data extracted from GS + and mapped by Excel https://www.microsoft.com/zh-cn/microsoft-365/excel.

Geostatistical analysis was conducted to analyze the spatial structure of variations in SAP and SAK. Table 4 shows the best geostatistical models that fit each variogram of SAP and SAK for the sampling sites, which has the lowest RSS values and highest R^2^ values. Figure 3 shows the semivariogram for the distribution of the SAP and SAK, where the solid line represents the best-fit model. Clearly, most variograms of SAP and SAK were fitted with an exponential model for all sampling sites (Table 4). In addition, Table 4 shows the spatial structure parameters, which are derived from the best-fitted models. Nugget values (C_0_) indicate undetectable experimental error and field variation within the minimum sampling space. Nugget values of SAP of Yangling, Changwu, Fuxian, Ansai and Shenmu were 3.41, 6.07, 14, 6.1 and 1.35, respectively, while values were 0.000001, 0.0001, 0.000001, 0.0003 and 0.0002, respectively, for SAK (Table 4). Clearly, the nugget values of SAP and SAK were low, especially for SAK. This result indicated that there were a small undetectable experimental error and field variation within the minimum sampling space. The Sill values (C_0_ + C) of SAP and SAK ranged from 5.71 to 49 and from 0.000001 to 0.001, respectively, which indicated the total spatial variation.

**Table 4:** Spatial structure parameters of soil available phosphorus (SAP) and soil available potassium (SAK) for the sampling sites.

SS	SP	Model	C_0_	C_0_ + C	C_0_/C_0_ + C	A_0_	RSS	R^2^
Yangling	SAP	Gaussian	3.41	10.3	0.33	71.01	0.8	0.98
	SAK	Spherical	0.000001	0.0005	0.002	1.89	4.413E−08	0.29
Changwu	SAP	Spherical	6.07	12.15	0.5	38.5	7.31	0.87
	SAK	Exponential	0.0001	0.001	0.11	5.97	6.233E−08	0.78
Fuxian	SAP	Exponential	14	49	0.29	322.5	64.8	0.78
	SAK	Exponential	0.000001	0.0008	0.0011	3.78	8.11E−08	0.63
Ansai	SAP	Exponential	6.1	14.84	0.41	59.7	4.26	0.94
	SAK	Exponential	0.0003	0.0007	0.48	18.06	3.816E−08	0.76
Shenmu	SAP	Exponential	1.35	5.71	0.24	473.1	2.48	0.44
	SAK	Spherical	0.0002	0.00047	0.45	14.57	4.423E−08	0.66

*SS* sampling sites, *SP* structure parameters, *C*_*0*_ nugget, *C*_*0*_ + *C* sill, *C*_*0*_*/(C*_*0*_ + *C)* anisotropy ratio, *A*_*0*_ range, *RSS* residual sum of squares, *R*^*2*^ coefficient of determination.

The value of C_0_/(C + C_0_) shows the degree of spatial dependence. For SAP, the value of C_0_/(C + C_0_) ranged from 0.29 to 0.5 at the Yangling, Changwu, Fuxian, Ansai sampling sites and showed a moderate spatial dependence (except for Shenmu) according to the criterion of Cambardella et al.^18^. For SAK, Ansai and Shenmu showed moderate spatial dependence, while the rest showed strong spatial dependence. The spatial dependence of SAP and SAK was influenced by many factors, including intrinsic factors (soil texture, mineralogy, etc.) and extrinsic factors (soil fertilization, human activities, etc.), leading to different spatial dependence of SAP and SAK. The range was used to assess the correlated distance of soil properties, and the ranges of SAP and SAK ranged from 38.50 m to 473.10 m and from 1.89 m to 18.06 m, respectively. This result indicated that our sampling density was adequate for detecting the spatial autocorrelation distance of SAP and SAK.

## Discussion

### Effect on the formation of SAP and SAK on the Loess Plateau

The content of SAP all showed a downward trend at the five sampling sites. SAP is influenced by many factors, including the soil parent material, geographical position, and soil physical and chemical properties^19^. Element P of the deep profile mainly comes from the release of apatite on the Loess Plateau. The bedrock is covered by three loess layers, including Wucheng Loess, Lishi Loess, and Malan Loess on the Loess Plateau. Liu et al.^20^ found that the mean P content of Wucheng loess and the lower part of Lishi loess were lower than that of the upper part of Lishi loess and Malan loess, leading to a decreasing trend in element P. Thus, SAP showed a decreasing trend. We also found that SAK tended to increase at all of the sampling sites along with the profile (Fig. 2). Additionally, SAK was also influenced by many factors, but clay was the key factor that affected the variations because clay has a high adsorption capacity for SAK^21^. Furthermore, the clay content increased with the depth at all of the sampling sites (data not shown). Liu et al.^13^ also found that clay content showed an increasing trend by measuring the soil particle compositions for the typical Luochuan loess profile, thereby leading to an increasing trend in SAK.

The mean SAP and SAK contents tended to decrease from south to north, that is, from Yangling to Changwu, Fuxian, Ansai, and Shenmu. Two reasons may explain the changes in the SAP concentration, as follows: (1) The temperature and precipitation increased from north to south among the sampling sites, and thus, P was more readily weathered and released from rocks^22^, thereby leading to an increase in the soil total P from north to south^23^. In addition, there was a positive correlation between the STP and SAP contents^13^. Therefore, the SAP contents tended to decrease from south to north among the sampling sites. (2) In addition to plants and fertilization, the soil texture is an important factor that influences the variations in the SAP contents in the deep profile. Soil with a higher adsorption capacity has a greater clay content^19^ and^24^ showed that clay has a higher SAP content compared to sand. We found that the clay content tended to decrease from south to north among the sampling sites, and thus, SAP content was higher in the south. SAK is also influenced by many factors, such as cultivation, soil texture, pH, and temperature. Therefore, given the depths of the sampling sites, the soil texture is an important factor that influences the SAK concentration because the clay content has important effects on the adsorption of SAK^21^. We found that the mean clay content tended to decrease among the sampling sites from south to north, and thus the mean SAK content decreased from south to north.

### Relationship between soil properties and SAP and SAK

The analysis was conducted based on the Pearson’s correlation coefficients to determine the strengths of the possible relationships between SAP and SAK, and their relationships with other soil properties. The correlation analysis results obtained for SAP varied among the different sampling sites. In addition, SMLRA showed the adjusted r^2^ values were low for SAP (except at Fuxian), thereby indicating that a complex array of factors influenced the SAP contents^13,19^. The results were also different for SAK, but the SAK contents determined at all of the sampling sites had significant positive correlations with the depth and clay content, while there was a significant negative correlation with the sand content. In addition, SMLRA of SAK showed the adjusted r^2^ values were high, and then depth and clay content were important variables to explain most of the variation in the SAK contents. Thus, clay was an important variable for SAK. In addition, depth is also an important variable for SAK variation because depth showed an increasing trend, as well as also an increasing trend for SAK, leading to a significant relationship between depth and SAK.

## Conclusions

In this study, we investigated five typical sampling sites on the Loess Plateau, China, and determined the spatial variations in the SAP and SAK from the top of the soil profile down to the bedrock. SAP showed an increasing trend along with the profile at the five sites and a decreasing trend for SAK. The mean SAP and SAK contents tended to decrease from south to north among the sampling sites. Clay and depth were important variables for explaining the variations in SAK. The mechanism responsible for the variations in the SAP content is complex, and many variables need to be considered to understand these changes. The exponential model could fit most variograms of SAP and SAK for all sampling sites. Due to the influence of many factors, such as human activities, soil texture and other factors, SAP and SAK at different sampling sites showed different spatial correlations. Our study is a basic study on the content changes of soil SAP and SAK in the deep profile of Loess Plateau, which can provide new ideas for the investigation of the biogeochemical cycle.
